# Ethanol production from a biomass mixture of furfural residues with green liquor-peroxide saccarified cassava liquid

**DOI:** 10.1186/s12896-016-0278-5

**Published:** 2016-06-01

**Authors:** Li Ji, Tianran Zheng, Pengxiang Zhao, Weiming Zhang, Jianxin Jiang

**Affiliations:** Department of Chemistry and Chemical Engineering, MOE Engineering Research Center of Forestry Biomass Materials and Bioenergy, Beijing Forestry University, Beijing, 100083 China; State Grid Energy Conservation Service CO., LTD. Beijing Biomass Energy Technology Center, Beijing, 100052 China; Nanjing Institute for the Comprehensive Utilization of Wild Plant, Nanjing, 210042 China; No. 35 East Qinghua Road, Haidian District Beijing, 100083 People’s Republic of China

**Keywords:** Ethanol, Cassava residues, Furfural residues, Green liquor-hydrogen peroxide (GL-H_2_O_2_)

## Abstract

**Background:**

As the most abundant renewable resources, lignocellulosic materials are ideal candidates as alternative feedstock for bioethanol production. Cassava residues (CR) are byproducts of the cassava starch industry which can be mixed with lignocellulosic materials for ethanol production. The presence of lignin in lignocellulosic substrates can inhibit saccharification by reducing the cellulase activity. Simultaneous saccharification and fermentation (SSF) of furfural residues (FR) pretreated with green liquor and hydrogen peroxide (GL-H_2_O_2_) with CR saccharification liquid was investigated. The final ethanol concentration, yield, initial rate, number of live yeast cells, and the dead yeast ratio were compared to evaluate the effectiveness of combining delignificated lignocellulosic substrates and starchy substrates for ethanol production.

**Results:**

Our results indicate that 42.0 % of FR lignin removal was achieved on FR using of 0.06 g H_2_O_2_/g-substrate and 9 mL GL/g-substrate at 80 °C. The highest overall ethanol yield was 93.6 % of the theoretical. When the ratio of 0.06 g/g-H_2_O_2_-GL-pretreated FR to CR was 5:1, the ethanol concentration was the same with that ratio of untreated FR to CR of 1:1. Using 0.06 g/g-H_2_O_2_-GL-pretreated FR with CR at a ratio of 2:1 resulted in 51.9 g/L ethanol concentration. Moreover, FR pretreated with GL-H_2_O_2_ decreased the concentration of byproducts in SSF compared with that obtained in the previous study.

**Conclusions:**

The lignin in FR would inhibit enzyme activity and GL-H_2_O_2_ is an advantageous pretreatment method to treat FR and high intensity of FR pretreatment increased the final ethanol concentration. The efficiency of ethanol fermentation of was improved when delignification increased. GL-H_2_O_2_ is an advantageous pretreatment method to treat FR. As the pretreatment dosage of GL-H_2_O_2_ on FR increased, the proportion of lignocellulosic substrates was enhanced in the SSF of the substrate mixture of CR and FR as compared with untreated FR. Moreover, the final ethanol concentration was increased with a high ethanol yield and lower byproduct concentrations.

## Background

There is currently an upsurge of interest in the search for renewable biomass to produce liquid transportation fuels such as bioethanol. This interest has been resulted from environmental concerns about toxic-gas emissions from burning petroleum fuels as well as a decrease in the available petroleum resources and fossil fuels. Increased attention has been paid on bioethanol as a promising alternative energy source [1]. Bioethanol is the most important biofuel today, which accounts for more than 90 % of total biofuel use [2].

Increasing attention is paid on ethanol production from lignocellulosic materials, especially low-cost waste materials [3], due to the abundance of lignocellulosic materials among the renewable resources. FR is an industrial byproduct from corncobs-based furfural production [4]. Furfural production process involves treating corncobs under heated and acidic conditions (hemicelluloses) [5]. The resultant FR is composed mainly of cellulose and lignin [6]. Cellulose and lignin in the cobs are relatively stable under furfural production conditions. Therefore, FR could be a great candidate material for ethanol production after pretreatment. Utilization of FR to produce ethanol would be effective in the dispose of waste products, while reducing environmental pollution.

It has been previously reported that the presence of lignin in biomass substrates might inhibit enzymatic hydrolysis [7], as the interaction between the lignin and the enzyme would reduce the activity of cellulase. The conversion of fermentable sugars is a crucial process for bioethanol production [8, 9]. Therefore, biomass delignification is an efficient approach for ethanol production to increase the available surface area of cellulose, dissolve the lignin, and break the bonds between lignin and carbohydrate polymers, and finally dissolve the lignin. As for FR, alkaline hydrogen peroxide pretreatment could effectively degrade the acidic lignin [10].

Green liquor (GL), which is an alkaline mixture of sodium carbonate and sodium hydroxide, can be used in alkaline pretreatment, and it has recently been developed for biomass applications [11]. This method could selectively remove lignin and recover cellulose efficiently [12]. The previous study showed that there was 56.7 % lignin removal ratio using alkaline hydrogen peroxide pretreatment [10]. In the present study, GL and hydrogen peroxide (GL-H_2_O_2_) were combined as a pretreatment for FR delignification with the addition of ethylenediaminetetraacetic acid (EDTA), which is a heavy metal ion chelate effective in reducing H_2_O_2_ degradation [13].

Cassava residues (CR) are dry solid fibrous byproducts of the starch industry [14], with an annual production of approximately 30 thousand tons in China [13]. CR is composed of starch (45–60 %), cellulose (15–25 %), and hemicellulose (<5 %) [15]. Due to the low cellulose content and high starch content, CR might be considered as a cellulosic starch byproduct [16] that could be easily processed using starch ethanol techniques into several valuable products. In addition, CR contains a certain amount of protein, which could provide the necessary nutrients for yeast fermentation. However, low ethanol yields have been reported with CR due to the poor accessibility of the CR slurry to cellulolytic enzymes [17]. It is more advantageous to combine CR with lignocellulosic material for use in ethanol production [5].

With a high ethanol concentration in the fermentation broth, the energy consumption of distillation could be reduced. A higher sugar concentration can be achieved by using a higher substrate concentration during the process of lignocellulose enzymatic hydrolisis. However, this can also lead to a higher concentration of inhibitors and when the whole slurry is used it may lead to a decreased yield as a result of inhibition such as by glycerol and due to poor mass transfer [18]. Recently, methods have been developed for efficient hydrolysis and fermentation of mixed fermentative microorganisms [19, 20]. Erdei et al. [21] developed a simultaneous saccharification and fermentation (SSF) process for wheat straw and wheat meal and illustrated that SSF of these mixed substrates could enhance ethanol production. Using substrate mixtures is promising to increase final ethanol concentrations and to replace starch with lignocelluloses. One possibility to produce a high final yield in SSF of mixtures is the dilution of inhibitors. Brandberg et al. [22] showed that starch hydrolysates are potential supplements for ethanol production from lignocellulosic hydrolysates. In the present work, the SSF of FR, which was subjected to GL-H_2_O_2_ pretreatment (GL-H_2_O_2_-FR), and CR, were carried out. The final ethanol concentration, yield, concentrations of byproducts, number of live yeast cells, and the dead yeast ratio were compared.

## Results and discussion

### Effects of H_2_O_2_ concentration on the chemical composition of pretreated FR

**Fig. 1:**
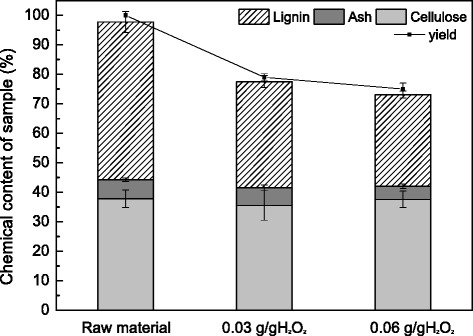
The effects of hydrogen peroxide concentration on the chemical composition of GL-H_2_O_2_ pretreated FR. Raw material represents untreated FR, while 0.06 g/g H_2_O_2_ represents FR pretreated with high loading of 0.06 g H_2_O_2_/g-substrate and 9 mL GL/g-substrate at 80 °C and 0.03 g/g H_2_O_2_ represents FR pretreated with high loading of 0.03 g H_2_O_2_/g-substrate and 9 mL GL/g-substrate at 80 °C

Untreated FR contained 37.8 % cellulose, 53.5 % acid insoluble lignin, and 6.5 % ash. The lignin content was higher than that of Yu et al. [4] because of the different batches of FR from factory. Cellulose and lignin are the main components, accounting production step with the addition of acid [23]. FR was pretreated with various concentrations of H_2_O_2_, as shown in Fig. 1, with 9 mL GL/g-dry substrate for 3 h at 80 °C and pH 12.0. After the GL-H_2_O_2_ pretreatment, the solid yield was reduced with increasing amounts of H_2_O_2_ (from 0.03 to 0.06 g/g-dry substrate), ranging from 79.0 to 75.0 % (Fig. 1). Likewise, the content of lignin decreased (Fig. 1). Approximately 42.0 % of the lignin was removed using 0.06 g H_2_O_2_/g-substrate, whereas 32.7 % was removed using 0.03 g H_2_O_2_/g-substrate. Unlike the lignin, 74.1 and 74.6 % of cellulose recovery were obtained from the substrate pretreated with 0.03 and 0.06 g H_2_O_2_/g-substrate, respectively (Fig. 1). Alkaline solutions have classically been used to bleach wood pulp [24], and the pretreatment’s efficacy due to alkali hydrogen peroxide is higher than alkaline alone for FR pretreatment [25]. Under these conditions, lignin would be decomposed to a low molecular weight and soluble oxidation product. In addition, Fe^2+^ present in the GL could react with hydrogen peroxide. In order to reduce the degradation of H_2_O_2_, EDTA was added in the pretreatment to form a complex with Fe^2+^. The pretreatment conditions were relatively mild for FR cellulose, with the mentioned sodium carbonate and sodium hydroxide content, thus the cellulose was not significantly degraded during the GL-H_2_O_2_ pretreatment.

### Ethanol and glucose concentrations during SSF with various mixture ratios of pretreated FR and CR

**Fig. 2:**
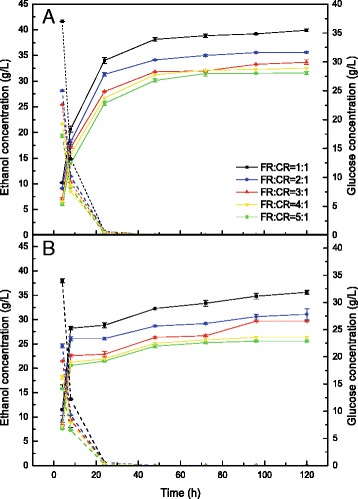
Concentrations of ethanol (*solid line*) and glucose (*dashed line*) during simultaneous saccharification and fermentation (SSF) of pretreated furfural residues (FR) and cassava residues (CR) of different mixture ratios with 12 % substrate loading at pH 6 and 38 °C. **a** represents the mixed substrates of 0.06 g/g-H_2_O_2_-GL-pretreated FR and CR, **b** represents the mixed substrates of 0.03 g/g-H_2_O_2_-GL-pretreated FR and CR

The composition of GL-H_2_O_2_-pretreated FR is shown in Fig. 1, and CR contains 47.2 % starch, 25.0 % cellulose, 3.7 % protein, and 6.8 % acid insoluble lignin. The GL-H_2_O_2_-pretreated FR with two levels of H_2_O_2_ treatments was used for SSF in five different ratios with 12 % substrate loading. Figure 2 shows the concentrations of glucose and ethanol as a function of time during SSF. Different ratios of GL-H_2_O_2_-pretreated FR and CR exhibited similar trends for glucose release, but there was a gradual decline of glucose level with an increase in GL-H_2_O_2_-pretreated FR to CR ratios (Fig. 2). As shown in Fig. 2, higher amounts of glucose were obtained at each sampling point for CR mixtures with GL-H_2_O_2_-pretreated FR with higher peroxide content (0.06 g/g-dry substrate H_2_O_2_) compared with mixtures of GL-H_2_O_2_-pretreated FR with lower peroxide content (0.03 g/g-dry substrate H_2_O_2_). However, glucose was almost completely consumed within 24 h, under the various conditions of SSF (Fig. 2).

**Fig. 3:**
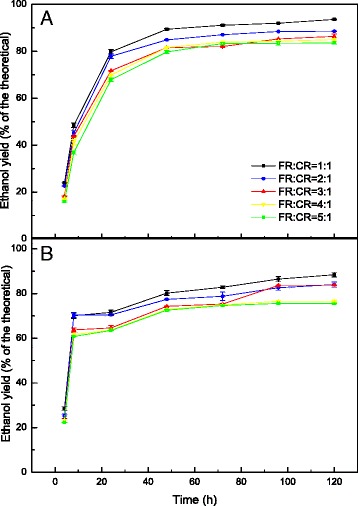
Ethanol yield of the theoretical during simultaneous saccharification and fermentation (SSF) of pretreated furfural residues (FR) and cassava residues (CR) of different mixture ratios with 12 % substrate loading at pH 6 and 38 °C. **a** represents the mixed substrates of 0.06 g/g-H_2_O_2_-GL-pretreated FR and CR, **b** represents the mixed substrates of 0.03 g/g-H_2_O_2_-GL-pretreated FR and CR

The mixtures of 0.06 g/g-H_2_O_2_-GL-pretreated FR and CR reached a concentration of over 30 g/L of ethanol in all five groups. 39.9 g/L of ethanol concentration was obtained using a 1:1 ratio of 0.06 g/g-H_2_O_2_-GL-pretreated FR to CR (Fig. 2a), which corresponds to an ethanol yield of approximately 93.6 % of the theoretical yield (Fig. 3a). Both ethanol concentration and yield decreased with increasing amounts of 0.06 g/g-H_2_O_2_-GL-pretreated FR. A concentration of 31.6 g/L of ethanol was achieved when the ratio of 0.06 g/g-H_2_O_2_-GL-pretreated FR to CR was increased to 5:1 (Fig. 2a), and the ethanol yield was 83.5 % of the theoretical yield (Fig. 3a). A significant increase in both ethanol concentration and yield occurred with the addition of CR. As mentioned above, CR contains 3.7 % protein and can act as a source of nitrogen, which is a necessary nutrient for ethanol production. Decreasing lignin had a positive impact on ethanol production in the experiment (Fig. 3). This is potentially the result of decreasing the adsorbed effect of lignin on cellulase.

As shown in Fig. 2b, more than 25 g/L of ethanol was obtained using a mixture of 0.03 g/g-H_2_O_2_-GL-pretreated FR and CR, and the highest ethanol concentration was 35.6 g/L with an ethanol yield of 88.4 % of the theoretical yield when the ratio was 1:1 (Fig. 3b). The lowest ethanol concentration occurred at a mixture ratio of 5:1 with an ethanol concentration of 25.5 g/L and an ethanol yield of 75.4 % of the theoretical yield.

There were similar results for ethanol concentration in SSF when the ratio of 0.06 g/g-H_2_O_2_-GL-pretreated FR to CR was 5:1 and the 0.03 g/g-H_2_O_2_-GL-pretreated FR to CR ratio was 3:1 (Fig. 2). For untreated FR, the ethanol concentration could reach the similar level under the FR:CR ratio of 1:2, (data not shown). GL-H_2_O_2_ pretreatment has a positive impact on FR, as the proportion of FR in SSF of the CR and FR mixture was enhanced after pretreatment with GL-H_2_O_2_ compared with untreated FR [15]. Thus, decreasing the content of lignin within FR using GL-H_2_O_2_ pretreatment improved the material usage. CR promotes FR by providing important nutrients for fermentative organisms, and in turn when SSF is performed with the substrate of FR as well as CR, it is necessary to add cellulase, and they hydrolyse the cellulosic fraction in CR for additional ethanol. Tang et al. [5] showed that SSF of FR alone with mineral-salt medium (without yeast extract) exhibited ethanol yield lower than that with organic medium, and also lower than that of mixture substrates with corn kernels. The organic nutrients were crucial for SSF of FR.

Table 1 shows the results of batch fermentations that are conducted at a pilot scale. The initial ethanol production was 2.88 g⋅L^−1^⋅h^−1^ when the ratio of 0.03 g/g-H_2_O_2_-GL-pretreated FR to CR was 1:1, which decreased to 1.89 g⋅L^−1^⋅h^−1^ with a ratio of 5:1. In the initial phase of fermentation, the production rate of ethanol from 0.03 g/g-H_2_O_2_-GL-pretreated FR and CR was higher than that from 0.06 g/g-H_2_O_2_-GL-pretreated FR and CR. However, the ethanol production rate after 120 h of SSF showed the opposite trend. There could be more inhibitors released in the initial phase of SSF for 0.06 g/g-H_2_O_2_-GL-pretreated FR than for 0.03 g/g-H_2_O_2_-GL-pretreated FR. The addition of CR appeared to attenuate this effect. This was likely due to the dilution of the lignocellulosic stream, resulting in a lower concentration of inhibitors [26]. Similar result has been reported previously [15]. The ethanol concentration was 36.2 g/L and the ethanol yield was 71.1 % of the theoretical yield with untreated FR plus CR at a ratio of 1:2. The similar concentrations and lower ethanol yields with increasing amounts of FR were indicative of lignin inhibition of ethanol production [23].

**Table 1:** The effects of substrate mixture ratios on final ethanol yield (% of the theoretical) and the initial rates of products before 4 h

Mixture ratio	Glucose release	Ethanol	Glycerol	Lactic acid	Acetic acid	Ethanol yield
	g/(L · h)	g/(L · h)	g/(L · h)	g/(L · h)	g/(L · h)	%
0.06 g/g-H_2_O_2_-GL-pretreated FR: CR						
1:1	9.25	2.55	0.18	0	0	93.61
2:1	6.25	2.28	0.21	0	0	89.86
3:1	5.65	1.78	0.23	0	0	87.28
4:1	4.83	1.64	0.28	0	0	87.13
5:1	4.30	1.51	0.34	0	0	83.50
0.03 g/g-H_2_O_2_-GL-pretreated FR: CR						
1:1	8.50	2.88	0.56	0	0	88.40
2:1	5.50	2.33	0.64	0	0	87.67
3:1	4.80	2.10	0.70	0	0	86.86
4:1	4.05	2.02	0.75	0	0.06	78.88
5:1	3.53	1.89	0.84	0	0.12	75.40

### Byproducts in SSF of different mixture ratios of pretreated FR and CR

**Fig. 4:**
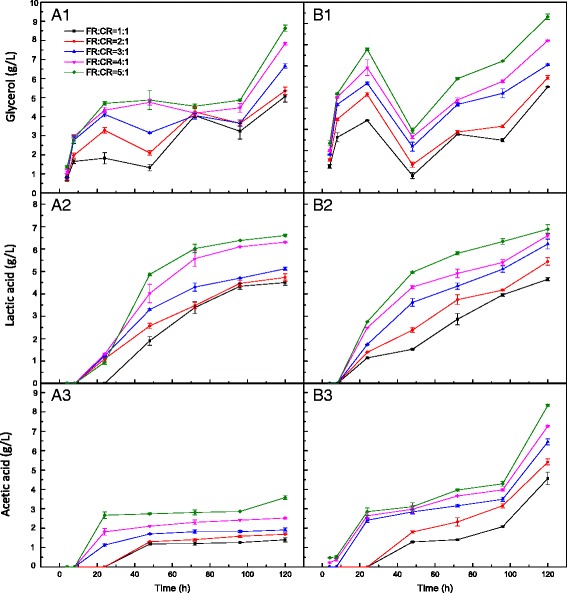
Concentrations of byproducts during simultaneous saccharification and fermentation (SSF) of pretreated furfural residues (FR) and cassava residues (CR) of different mixture ratios with 12 % substrate concentration at pH 6 and 38 °C. A1, A2, A3 represent the mixed substrates of 0.06 g/g-H_2_O_2_-GL-pretreated FR and CR, B1, B2, B3 represent the mixed substrates of 0.06 g/g-H_2_O_2_-GL-pretreated FR and CR

Raw material costs are themselves considerable (approximately 30 % on an annual basis), and utilization of available biomass is important [27]. Pretreated FR (with the two peroxide concentration level GL-H_2_O_2_ treatments, 0.06 g/g-H_2_O_2_-GL-pretreated FR and 0.03 g/g-H_2_O_2_-GL-pretreated FR) was combined with CR at various ratios, and the SSF efficiencies were measured and compared with each other. Figure 4a illustrates the byproducts produced by SSF carried out with mixtures of 0.06 g/g-H_2_O_2_-GL-pretreated FR and CR, and Fig. 4b is that of 0.03 g/g-H_2_O_2_-GL-pretreated FR in combination with CR. The investigated concentration range of these chemicals was chosen to examine possible applications involving concentrated slurries.

#### Glycerol

In sample A (Fig. 4A1), the highest glycerol concentration (8.6 ± 0.16 g/L) was formed at 120 h with a ratio of 0.06 g/g-H_2_O_2_-GL-pretreated FR to CR of 5:1, while in sample B it reached 9.3 ± 0.13 g/L with the same ratio (Fig. 4B1). Glycerol acts as an inhibitor of SSF as it consumes at least 4 % of the carbon source for the fermentation [15]. There was a high concentration of glycerol at 24 h, ranging from 1.8 to 4.7 g/L, with an increase of the mixture substrate ratio of 0.06 g/g-H_2_O_2_-GL-pretreated FR to CR from 1:1 to 5:1 (Fig. 4A1); however, it ranged from 4.4 to 7.8 g/L for the mixture of 0.03 g/g-H_2_O_2_-GL-pretreated FR and CR (Fig. 4B1). A decrease in glycerol concentration occurred at 48 h, which is likely the result of the conversion of glycerol into dihydroxyacetone [28]. When half pretreated FR (either 0.06 g/g or 0.03 g/g-H_2_O_2_-GL-pretreated FR) and half CR was used in a mixture, the highest glycerol concentrations were 5.1 to 6.0 g/L, while 8.3 g/L was reached using untreated FR plus CR with the same ratio (data not shown). These results indicate that delignification of the source material used for SSF could produce less glycerol byproducts under the same conditions. In addition, glycerol would be against the osmotic pressure and would reduce the oxidation-reduction potential [29], which would result in the concentration fluctuation (Fig. 4). Yeast cells lack acetaldehyde as a hydrogen acceptor, which leads to increased NADH as concentration is increased. NAD^+^ required for xylitol dehydrogenase (XDH), can be regenerated by reducing dihydroxyacetone phosphate to glycerol [30].

#### Lactic acid (LA)

The increase in the production of LA would accompany a decrease in the final ethanol concentration [31]. Figure 4 shows that little LA was produced during the first 8 h for both levels of H_2_O_2_ pretreated FR. For mixtures containing a ratio of 0.06 g/g-H_2_O_2_-GL-pretreated FR to CR of 1:1, LA production occurred after 24 h. The production of LA was delayed in 0.06 g/g-H_2_O_2_-GL-pretreated FR compared with 0.03 g/g-H_2_O_2_-GL-pretreated FR. This could be due to the high delignification that occurred with 0.06 g/g-H_2_O_2_-GL-pretreated FR leading to a lower concentration of LA [32]. The overall trend of the LA concentration was increasing with increasing proportion of pretreated FR. However, the final LA concentration for the mixture ratios of 2:1 and 1:1 were quite similar. The reduced LA concentration was observed when more CR was used in the substrate mixture. Besides that, the absence of LA with increased pretreatment of FR indicates that the degraded lignin in the pretreatment liquid inhibit LA bacteria [33]. Moreover, increased concentrations of CR provide more nutrients, which decrease the production of LA in both cases [5].

#### Acetic acid

In SSF of mixtures of 0.06 g/g-H_2_O_2_-GL-pretreated FR and CR, the acetic acid concentration at 24 h was 1.1 g/L, 1.8 g/L, and 2.7 g/L for mixture have a ratio of 3:1, 4:1, and 5:1, respectively (Fig. 4A3). However, in SSF with mixture having a ratio of 1:1 or 2:1, there was no acetic acid production prior to 24 h. On the other hand, 0.03 g/g-H_2_O_2_-GL-pretreated FR mixtures with CR incurred higher concentrations of acetic acid (Fig. 4B3). The addition of CR delayed the production of acetic acid; at 48 h, for mixtures with ratio of 1:1 and 2:1, the acetic acid concentration was 1.3 g/L and 1.8 g/L, respectively. Acetic acid concentrations more than 2 g/L would reduce ethanolic fermentation [27]. The acetic acid concentration of the mixtures of 0.03 g/g-H_2_O_2_-GL-pretreated FR and CR was twice that of the mixtures of 0.06 g/g-H_2_O_2_-GL-pretreated FR and CR, indicating that FR delignification has a positive effect on lowering acetic acid production. The formation of acetic acid is a kind of metabolism emanation [34]. Hanmilton considered that the inhibition of acetic acid growth was not only the release of proton but also the accumulation of the anion. The inhibition of acetic acid formation was the comprehensive results [35].

The initial rate of byproduct production in SSF is shown in Table 1. Both the concentration and initial production rate of glycerol and acetic acid were increased with increasing addition of pretreated FR or by reducing the pretreatment intensity (or level of peroxide), resulting in lower ethanol production. An additional benefit of substrate delignification for ethanol production is clearly reflected in the current study. When the pretreated substrate is added to the medium, it reduces the effect of the inhibitors.

### Ethanol and glucose concentrations of SSF with different substrate loadings

**Fig. 5:**
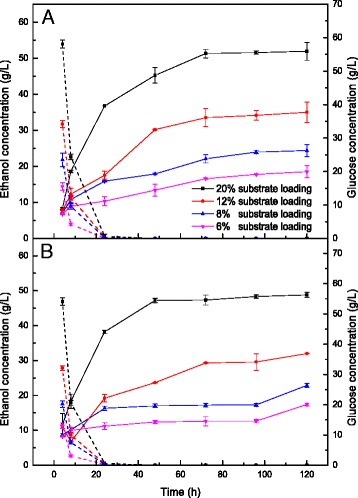
Concentrations of ethanol (*solid line*) and glucose (*dashed line*) during simultaneous saccharification and fermentation (SSF) of furfural residues (FR) and cassava residues (CR) of different substrate loadings with an FR:CR ratio of 2:1 at pH 6 and 38 °C. **a** represents the mixed substrates of 0.06 g/g-H_2_O_2_-GL-pretreated FR and CR, **b** represents the mixed substrates of 0.03 g/g-H_2_O_2_-GL-pretreated FR and CR

The ethanol concentration reached 51.9 g/L for 20 % substrate loading with a mixture of 0.06 g/g-H_2_O_2_-GL-pretreated FR and CR (Fig. 5a), making for an ethanol yield of 79.2 % (data not shown). The trends of ethanol production in the presence of lignin degradation are similar to that discussed above with pretreated FR and CR at various ratios. For the entire range of concentrations explored, no glucose was detected after 24 h. In contrast, the ethanol concentration decreased to 48.8 g/L for 20 % substrate loading with a mixture of 0.03 g/g-H_2_O_2_-GL-pretreated FR and CR (Fig. 5b), and the corresponding ethanol yield was of approximately 79.0 %. There was an observed downward trend in the final ethanol concentration after 120 h with decreasing substrate concentration or FR pretreatment intensity. The ethanol concentration value increased during the first 48 h and then remained unchanged until after 72 h for both sample A and sample B (Fig. 5). The ethanol yield decreased with increasing substrate concentration (data not shown), likely the result of higher lignocellulosic substrate viscosity leading to higher concentrations of inhibitors [18]. Delignification of the raw materials had clear effects on SSF for both ethanol concentration and ethanol yield. The ethanol concentration increased by 27.8 % between 0.06 g/g-H_2_O_2_-GL-pretreated FR and untreated FR at the same substrate concentration [15].

Another potential explanation for the increase in ethanol production when using CR might be the supplemented nutrient content. It has been shown that wheat hydrolysate can sustain anaerobic cultivation of *Saccharomyces cerevisiae* [22]. In addition, Tang et al. illustrated that corn hydrolysate can be used for the replacement of the organic medium in SSF of FR, as it is a complex organic nutrient [5]. The additional benefits of the amount of CR in the medium is clearly reflected in the current study.

### The number of live yeast cells and the dead yeast ratio during SSF of pretreated FR mixed with CR

**Fig. 6:**
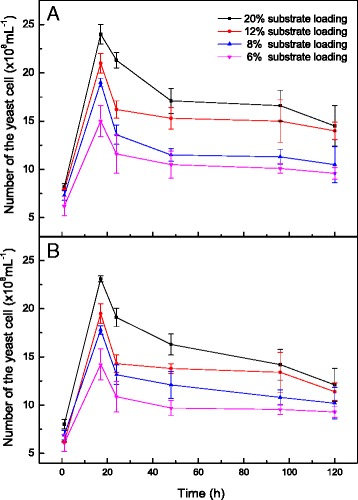
The number of live yeast cells during simultaneous saccharification and fermentation (SSF) of furfural residues (FR) and cassava residues (CR) of different substrate loadings with an FR:CR ratio of 2:1 at pH 6 and 38 °C. **a** represents the mixed substrates of 0.06 g/g-H_2_O_2_-GL-pretreated FR and CR, **b** represents the mixed substrates of 0.03 g/g-H_2_O_2_-GL-pretreated FR and CR

The profile of yeast concentration is shown in Fig. 6. When fermentation was conducted with FR and CR at 6 % substrate loading, the exponential and stationary phases were prolonged with a low-level phase, which indicates that yeast cell metabolism and growth decreased at the 6 % substrate concentration (Fig. 6). Higher substrate concentrations would provide an increased source of carbon [36], which affects the yeast growth rate [5]. Tang et al. [37] explained that temperature affects carbon uptake, indicating that yeast cell concentration can be influenced by different factors. The yeast cell concentration was increased in SSF of mixtures of 0.06 g/g-H_2_O_2_-GL-pretreated FR and CR (Fig. 6a). The maximum cell concentration achieved at the 20 % substrate concentration, which suggests that increasing nutrient concentration could give rise to the increased yeast concentration during ethanol fermentation at 20 % substrate concentration compared with the lower substrate concentrations.

**Fig. 7:**
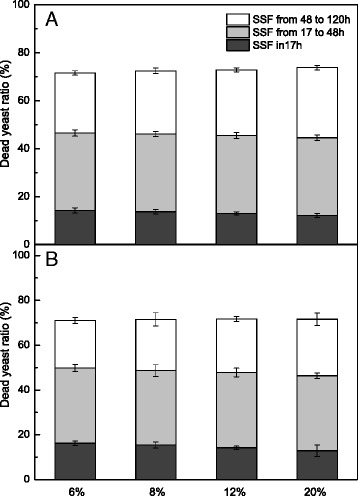
The number of dead yeast cells during simultaneous saccharification and fermentation (SSF) of furfural residues (FR) and cassava residues (CR) of different substrate loadings with an FR:CR ratio of 2:1 at pH 6 and 38 °C. **a** represents the mixed substrates of 0.06 g/g-H_2_O_2_-GL-pretreated FR and CR, **b** represents the mixed substrates of 0.03 g/g-H_2_O_2_-GL-pretreated FR and CR. 6 %, 8 %, 12 %, 20 % represent the substrate concentration, respectively

The dead cell ratio at 6 and 8 % substrate concentrations of 0.03 g/g-H_2_O_2_-GL-pretreated FR and CR was initially high, which explains the hysteretic stationary phase observed during fermentation with these mixtures (Fig. 7b). A similar dead cell ratio was observed at 6 and 8 % substrate concentration with mixtures of 0.06 g/g-H_2_O_2_-GL-pretreated FR and CR (Fig. 7a). Our results indicate that the dead cell ratio at 20 % substrate concentration was the lowest, but it increased with fermentation time. A high dead cell ratio represented increased sugar loss due to mortality of the yeast cells [37]. Ethanol fermentation occurs mainly in the first 17 h, during which the yeast cells grow and metabolize actively, generating a low dead yeast ratio. A higher dead cell ratio of 30 % was observed from 17 to 48 h due to a decreasing glucose concentration and increasing ethanol concentration. After 48 h, glucose was depleted and the ethanol concentration approached the maximum value, caused a massive die-off of yeast cells. Delignification has an effect on yeast growth. The mixtures of untreated FR and CR had a large variation among the different substrate concentrations, and they exhibited a lower cell concentration and higher dead cell ratio during the first 17 h of SSF [15].

## Conclusions

The results of this investigation indicated that using delignified FR combined with CR as a source of readily fermentable sugars represents an untapped source for ethanol biofuel production. Our results suggested that the increase in H_2_O_2_ concentration intensified the delignification of FR by increasing oxidation. The efficiency of ethanol fermentation was improved when delignification increased.

High intensity of FR pretreatment increased the final ethanol concentration, resulting in a high ethanol yield and a downward trend in the production of byproducts compared with SSF of the mixture of untreated FR and CR. The combination of GL-H_2_O_2_ pretreated FR and CR provided sufficient organic nutrients from starch materials for SSF and the production of additional ethanol from CR is beneficial for the integration of cellulosic and starch material, which leads to a high ethanol yield in SSF. Ethanol production from SSF of a high substrate concentration of GL-H_2_O_2_ pretreated FR and CR is therefore cost-efficient.

The ethanol concentration increased by 27.8 % from untreated FR to 0.06 g/g-H_2_O_2_-GL-pretreated FR under the same substrate loading conditions. The number of live yeast cells increased with increasing substrate concentration, while the dead yeast ratio decreased.

Further investigation is necessary to balance the ratio of GL-H_2_O_2_ pretreated FR and CR at high substrate concentration to improve the final ethanol concentration.

## Methods

### Raw material

Raw FR was kindly provided by Chunlei Company (Hebei Province China). Raw FR, with an initial pH value was dried at 50 °C for 12 h after rinsed with water to neutral pH to remove inhibitors, including furfural and 5-HMF [27]. The FR filtered through the 40 meshes was collected as the substrate for use in this study. CR was kindly provided by Guangxi Key Laboratory of Chemistry and Engineering of Forest Products (Nanning, China).

The raw GL was supplied by Chenming Group (Shandong, China). A supernatant was obtained by the precipitation the raw GL overnight. The contents of sodium hydroxide and sodium carbonate were tested according to TAPPI T6424 cm-00 [38]. Calcium and iron contents of GL were measured according to TAPPI T266 om-2 [39]. The main composition of GL was sodium carbonate reaching to 75.2 ± 0.25 g/L. The sodium hydroxide content was 23.04 ± 0.25 g/L. Other metal elements in GL such as iron and calcium were 1.14 ± 0.08 g/L and 0.39 ± 0.03 g/L, respectively [4].

### Pretreatment with green liquor and hydrogen peroxide

An aliquot of 5 g of FR was put into a polytetrafluoroethylene (PTFE) reactor with a volume of 200 mL. For each experiment, the FR slurry in water (4.2 %, *w/v*) containing the desired amounts of GL and H_2_O_2_ (30 %, Sinopharm Chemical reagent Beijing Co., Ltd). GL loading was 9 mL GL/g-dry substrate, and stabilizer of EDTA (98 %, Sinopharm Chemical reagent Beijing Co., Ltd) was of 1 % g/g-dry substrate [4]. Two different loadings of 10 % H_2_O_2_ were evaluated: 0.03 g/g-dry substrate and 0.06 g/g-dry substrate. After sealing, the pretreatment reactor was loaded into a large stainless steel tank fitted with PTFE. The system (PTFE reactor + stainless steel tank) was placed in a chamber with a shaft able to rotate at variable speeds. The system was heated at an average rate of 5 °C/min to reach a temperature of 80 °C. After 3 h, the system was rapidly cooled with tap-water. The insoluble residues were collected by filtration and washed with distilled water until neutral pH. Some of the washed samples were dried in an oven at 105 °C for 6 h to obtain the yield. The solid yield was calculated using the following equation:

$$ \mathrm{Solid}\kern0.5em \mathrm{yield}\kern0.5em \left(\%\right)=\frac{\mathrm{mass}\kern0.5em \mathrm{of}\kern0.5em \mathrm{pretreated}\kern0.75em \mathrm{dry}\kern0.5em \mathrm{solid}\kern0.75em \left(\mathrm{g}\right)}{\mathrm{mass}\kern0.5em \mathrm{of}\ \mathrm{untreated}\kern0.75em \mathrm{dry}\kern0.5em \mathrm{solid}\kern0.75em \left(\mathrm{g}\right)}\times 100 $$

The composition of GL-H_2_O_2_ pretreated FR based on the initial FR can be obtained by multiplying the yield by the percentage of each component of GL-H_2_O_2_ pretreated FR.

### CR-starch hydrolysis

CR-starch hydrolysis was performed by a two-step enzyme method in a 500 mL flask with 20 % dry matter. Briefly, CR was firstly liquefied at 85 °C for 2 h using commercial amylase enzyme at a loading of 150 u/g-CR to decrease the viscosity of CR [40]. Subsequent saccharification was conducted at 60 °C for 1 h by glucoamylase at a loading of 20 u/g-CR (pH 4.0). Incomplete saccharification was implemented to reduce high concentrations of sugar and osmotic pressure on yeast [41]. The pH of CR saccharification liquid was adjusted to 5.5 with 10 % sodium hydroxide before SSF.

### Microorganism, inoculum and enzyme preparation

The microorganism used for fermentation was *S. cerevisiaein*, the form of dry yeast (Angel Yeast Company Ltd, Yichang, China). Dry yeast was activated in a 2 % glucose solution at 36 °C for 15 min, then at 34 °C for 1 h before SSF. The α-amylase and glucoamylase (Aoboxing Universeen Bio-Tech Company Ltd, Beijing, China) were used for CR liquefaction and saccharification, respectively. Cellulase (Celluclast1.5 L) with an activity of 75 filter paper units (FPU)/ml and β-glucosidase (Novozyme 188) with an activity of 43.9 IU/ml enzyme preparations (both Novozymes A/S, Bagsvaerd, Denmark) were used for SSF. The enzyme loading of Celluclast 1.5 Land Novozyme 188 were 15 FPU and 17 IU pergram cellulose of dry substrate, respectively.

### Simultaneous saccharification and fermentation

The SSF experiments were performed in a 100-mL Erlenmeyer flask with a working volume of 60 mL with a loop trap containing sterile glycerol. There were two sections of this study: fixed substrate concentration (12 %) with various ratios (ranging from 1:1 to 5:1) of pretreated FR to CR and fixed ratio (2:1) of pretreated FR to CR with different substrate concentrations (6 to 12 %). The concentrations ranged from 2 to 6.7 % for CR and 3 to 13.3 % for GL-H_2_O_2_-FR. Each Erlenmeyer flask loaded with substrate and fermentation medium was sterilized separately (121 °C, 20 min). The enzymes, CR hydrolysate and yeast, with an initial inoculum concentration of 3.3 g/L were then added to the Erlenmeyer flask directly. SSF was conducted at 38 °C and 120 rpm with an initial pH of 5.5 in an air bath shaker.

### Analytical methods

The contents of cellulose and hemicellulose of materials were analyzed using the National Renewable Energy Laboratory (NREL) methods [42]. The acid insoluble lignin analysis of samples were carried out according to the TAPPI method [43]. The CR starch was determined according to McCleary’s method [44]. The yeast cell concentration and dead cell ratio were carried out by blood-count method [5]. Total ash was calculated after calcined in a muffle furnace at 500 °C. Nitrogen content was multiplying the nitrogen content by a factor of 6.25. The fermentation samples were filtered (pore size, 0.22-μm) to detect glucose, ethanol and byproducts. Ethanol, glucose and byproducts were analyzed by high performance liquid chromatography (HPLC) (Waters 2695e, USA) using an Aminex HPX-87H column (300 × 7.8 mm; Bio-Rad Laboratories, USA) at 65 °C and refractive index detection detector at 30 °C. The ethanol yield was calculated as follows [10]:

$$ \mathrm{Ethanol}\ \mathrm{yield}\ \left(\%\right)=\frac{\mathrm{mass}\ \mathrm{of}\ \mathrm{ethanol}\ \mathrm{concentration}\ \left(\mathrm{g}\right)}{0.5679\times \mathrm{mass}\ \mathrm{of}\ \left(\mathrm{F}\mathrm{R}\ \mathrm{cellulose}+\mathrm{C}\mathrm{R}\ \mathrm{cellulose}+\mathrm{C}\mathrm{R}\ \mathrm{starch}\right)\ \left(\mathrm{g}\right)}\times 100 $$

Glucose released was evaluated for the first 4 h and the equation was presented:

$$ \mathrm{Glucose}\kern0.75em \mathrm{release}\kern0.75em \left(\mathrm{g}\cdot {\mathrm{L}}^{\hbox{-} 1}\cdot {\mathrm{h}}^{\hbox{-} 1}\right)=\frac{\mathrm{Glucose}\kern0.75em \left(\mathrm{g}/\mathrm{L}\right)}{\ \mathrm{cumulative}\kern0.5em \mathrm{time}\kern0.75em \left(\mathrm{h}\right)} $$

## Abbreviations

SSF, simultaneous saccharification and fermentation; CR, cassava residue; FR, furfural residue; GL, green liquor; GL-H_2_O_2_, green liquor and hydrogen peroxide; 0.06 g/g-H_2_O_2_-GL-pretreated FR, FR pretreated with high loading of 0.06 g H_2_O_2_/g-substrate and 9 mL GL/g-substrate at 80 °C; WIS, water-insolible solids; 0.03 g/g-H_2_O_2_-GL-pretreated FR, FR pretreated with high loading of 0.03 g H_2_O_2_/g-substrate and 9 mL GL/g-substrate at 80 °C; PTFE, polytetrafluoroethylene; EDTA, ethylenediaminetraacetic acid; FPU, Filter paper units.
